# Evaluating Bias-Mitigated Predictive Models of Perinatal Mood and Anxiety Disorders

**DOI:** 10.1001/jamanetworkopen.2024.38152

**Published:** 2024-12-03

**Authors:** Emily F. Wong, Anil K. Saini, Eynav E. Accortt, Melissa S. Wong, Jason H. Moore, Tiffani J. Bright

**Affiliations:** 1 Department of Computational Biomedicine, Cedars-Sinai Medical Center, Los Angeles, California; 2 Department of Obstetrics and Gynecology, Cedars-Sinai Medical Center, Los Angeles, California

## Abstract

**Importance:**

Machine learning for augmented screening of perinatal mood and anxiety disorders (PMADs) requires thorough consideration of clinical biases embedded in electronic health records (EHRs) and rigorous evaluations of model performance.

**Objective:**

To mitigate bias in predictive models of PMADs trained on commonly available EHRs.

**Design, Setting, and Participants:**

This diagnostic study collected data as part of a quality improvement initiative from 2020 to 2023 at Cedars-Sinai Medical Center in Los Angeles, California. The study inclusion criteria were birthing patients aged 14 to 59 years with live birth records and admission to the postpartum unit or the maternal-fetal care unit after delivery.

**Exposure:**

Patient-reported race and ethnicity (7 levels) obtained through EHRs.

**Main Outcomes and Measures:**

Logistic regression, random forest, and extreme gradient boosting models were trained to predict 2 binary outcomes: moderate to high-risk (positive) screen assessed using the 9-item Patient Health Questionnaire (PHQ-9), and the Edinburgh Postnatal Depression Scale (EPDS). Each model was fitted with or without reweighing data during preprocessing and evaluated through repeated K-fold cross validation. In every iteration, each model was evaluated on its area under the receiver operating curve (AUROC) and on 2 fairness metrics: demographic parity (DP), and difference in false negatives between races and ethnicities (relative to non-Hispanic White patients).

**Results:**

Among 19 430 patients in this study, 1402 (7%) identified as African American or Black, 2371 (12%) as Asian American and Pacific Islander; 1842 (10%) as Hispanic White, 10 942 (56.3%) as non-Hispanic White, 606 (3%) as multiple races, 2146 (11%) as other (not further specified), and 121 (<1%) did not provide this information. The mean (SD) age was 34.1 (4.9) years, and all patients identified as female. Racial and ethnic minority patients were significantly more likely than non-Hispanic White patients to screen positive on both the PHQ-9 (odds ratio, 1.47 [95% CI, 1.23-1.77]) and the EPDS (odds ratio, 1.38 [95% CI, 1.20-1.57]). Mean AUROCs ranged from 0.610 to 0.635 without reweighing (baseline), and from 0.602 to 0.622 with reweighing. Baseline models predicted significantly greater prevalence of postpartum depression for patients who were not non-Hispanic White relative to those who were (mean DP, 0.238 [95% CI, 0.231-0.244]; *P* < .001) and displayed significantly lower false-negative rates (mean difference, −0.184 [95% CI, −0.195 to −0.174]; *P* < .001). Reweighing significantly reduced differences in DP (mean DP with reweighing, 0.022 [95% CI, 0.017-0.026]; *P* < .001) and false-negative rates (mean difference with reweighing, 0.018 [95% CI, 0.008-0.028]; *P* < .001) between racial and ethnic groups.

**Conclusions and Relevance:**

In this diagnostic study of predictive models of postpartum depression, clinical prediction models trained to predict psychometric screening results from commonly available EHRs achieved modest performance and were less likely to widen existing health disparities in PMAD diagnosis and potentially treatment. These findings suggest that is critical for researchers and physicians to consider their model design (eg, desired target and predictor variables) and evaluate model bias to minimize health disparities.

## Key Points

**Question:**

How accurate and fair are predictive models of postpartum depression, defined as screening above clinical cutoffs on psychometric screening tools, trained on commonly available electronic health records?

**Findings:**

In this diagnostic study assessing predictive models of postpartum depression of 19 430 patients, models performed modestly (mean areas under the receiver operating curves ranged from 0.602 to 0.635) and did not display the same historical biases against racial and ethnic minority groups (significantly higher positive screen rates and lower false-negative rates).

**Meaning:**

Findings suggest that it is critical for researchers and physicians to consider their model design (eg, desired target and predictor variables) and evaluate model bias to minimize health disparities.

## Teaser

This diagnostic study assesses whether predictive models of perinatal mood and anxiety disorders that consider model design and are trained on commonly available electronic health record data can mitigate clinical biases embedded in those records and minimize health disparities.

## Introduction

Perinatal mood and anxiety disorders (PMADs) encompass a range of mental health disorders, including major depressive disorder and various anxiety disorders, that occur during pregnancy or up to 1 year post partum and affect approximately 20% of birthing parents.^1^ Postpartum depression (PPD) is the most common PMAD, affecting approximately 13% of birthing parents in high-income countries^2^ and 20% of birthing parents in low-income and middle-income countries.^3^ Despite recent national recommendations and state laws,^4,5,6^ nearly 50% of PMADs are never identified. Fewer than 1 in 4 women in Los Angeles, California, receive PMAD screening or education during prenatal, postpartum, or well-child medical visits.^7^ Even when they receive screening, many women do not respond openly to these questions.^8,9,10^ The consequences of untreated PMAD are substantial, including inhibited child development.^11,12,13,14,15,16,17,18,19^ When severe, PPD can lead to suicide, one of the leading causes of perinatal death.^20^ As with all health conditions, early detection and prevention are key. However, comprehensive clinical interviews are a time-intensive process that can be conducted only by a licensed mental health professional.^21,22^ The diagnostic efficacy of more brief, inpatient screening procedures can be affected by varying psychometric properties,^23^ method of administration,^24,25^ stigma,^26^ and human error.^9^ Thus, better diagnostic and prognostic models are needed for early identification and referral to treatment.^9,22,27,28^

Women from racial and ethnic minority groups are at an increased risk of PMAD.^29,30,31^ PMAD prevalence rates in low-income and ethnic minority women, for example, are more than double the expected rate (approximately 13%),^2^ with prevalence rates as high as 38% for depression.^32^ However, women from racial and ethnic minority groups are less likely to be screened for systemic reasons (eg, access) and less likely to follow-up with care for reasons related to perceived stigma, cultural beliefs, culturally insensitive interventions, and lower standard of care.^33,34,35,36,37,38^ For example, non-Hispanic White women are more likely than Black and Latina women to initiate postpartum mental health care, receive follow-up care, and refill prescriptions.^33^ Prior studies have also shown that low-income urban Black women self-report lower severity of depressive symptoms on psychometric questionnaires^23,39,40,41,42^ despite equivalent diagnoses, yielding higher rates of false negatives when compared with other groups; recommendations following these results suggest lowering the standard cutoffs for positive screening.

Due to the many barriers and the complex etiology of psychiatric disorders,^22^ identification of patients at risk of PMAD poses a major challenge for public health officials and health care professionals. To address this challenge, recent research offers machine learning as a clinical tool to support more routine screening of PMAD by using information obtained from electronic health records (EHRs), which readily include many highly predictive factors,^43,44^ such as prior mental health diagnoses,^45^ gestational age,^46^ infant birth weight,^47^ method of delivery,^48^ and neonatal intensive care unit admission.^49^ While machine learning has the potential to democratize medicine,^50^ such models are inherently prone to reproducing existing medical biases present in EHRs.^51^ Therefore, it is paramount to assess and account for any existing biases in the machine learning process.

Given the persistent disparities in obtaining diagnosis and treatment, this study emphasizes that the choices of target and predictor variables are imperative to ensure that the model does not widen existing disparities by reproducing biases in the dataset, an issue first raised by Obermeyer et al.^52^ While prior work on predictive models of PMAD has achieved impressive performance (areas under the receiver operating curves [AUROCs] of 0.60 to 0.93), the target variable is often represented as logged *International Classification of Diseases* (*ICD*) codes (ie, official diagnoses) and pharmacy records,^43,44,53,54,55,56,57^ or prior psychometric screening results are required as a key predictor in the model.^44,58,59,60,61,62,63^ However, given that women from racial and ethnic minority groups are less likely to receive screening and treatment, such models are inherently susceptible to reproducing existing biases. For example, such models predict greater risk of PMAD for White women than for Black women, even after debiasing,^55^ reflecting disparities in obtaining official diagnoses and treatment. Similar to Obermeyer et al,^52^ such algorithmic predictions are not necessarily indicative of lower prevalence of PMAD among Black women, but likely indicative of lower access or greater discrimination^42^ and perceived stigma.^42^ (Indeed, non-Hispanic White patients in our study were more likely than Asian, Black, or Hispanic patients to have been prescribed psychotherapeutic drugs prior to delivery [eTable 1 in Supplement 1]). While there are disparities in psychometric responding, adjusting for lower self-reported severity (eg, lowering cutoff values) is more tractable than adjusting for lower rates of logged *ICD* codes and pharmacy records. Moreover, models that use prior psychometric screening as a primary predictor may be unusable for communities that are unlikely to receive routine symptom screening.

The current study evaluated the performance of machine learning models trained on commonly available EHR data (ie, did not require prior screening) to predict psychometric screening outcomes; the models were also evaluated for potential bias. We do not recommend replacing traditional psychometric screening with machine learning. Instead, we suggest that machine learning can augment traditional procedures to encourage more equitable and routine screening for PMAD. For example, predictive modeling does not require patients to answer potentially sensitive questions regarding their mental health status (eg, suicidal ideation) in the presence of family or during stressful events (eg, being admitted for an emergency cesarean delivery), as is commonly done in the current workflow,^8^ which may contribute to higher false-negative screen rates in racial and ethnic minority women.

To our knowledge, only 2 studies have used psychometric screening for PPD as the target variable without requiring prior psychometric screening as a key predictor. When using comparable information for training, Matsuo et al^64^ achieved modest performance on predicting the Patient Health Questionnaire 9 (PHQ-9) screening results trained on EHRs obtained from multiple birthing centers in Japan (AUROCs = 0.569 to 0.626) but did not evaluate algorithmic bias.^64^ Huang et al^65^ also achieved modest performance on predicting the Edinburgh Postnatal Depression Scale (EPDS) screening results from EHRs obtained from an urban academic hospital in Chicago, Illinois (best AUROC = 0.670).^65^ Huang et al^65^ concluded that algorithmic performance was biased against Black and Latina women despite these groups comprising the majority group in the dataset; algorithmic bias was defined as lower AUROC values for Black and Latina women relative to non-Hispanic White women. Last, all prior work that evaluated model bias was limited to 2 or 3 racial and ethnic groups. The present study extends the current literature by evaluating model performance between multiple racial and ethnic groups and provides a more rigorous evaluation of model performances through repeated K-fold cross validation in lieu of reporting results from a single training-validation-testing split, which may yield overly optimistic results of model performance.

## Methods

### Data Collection

The report for this diagnostic study adheres to the Transparent Reporting of a Multivariable Prediction Model for Individual Prognosis or Diagnosis for Artificial Intelligence (TRIPOD + AI) guideline for reporting clinical prediction models. The data were collected between 2020 and 2023 as part of the Postpartum Depression Screening, Education and Referral Quality Improvement Initiative at Cedars-Sinai Medical Center (CSMC), located in Los Angeles, California. Since 2017, CSMC has routinely screened all patients with a live birth for PPD immediately following delivery. The aim of the quality improvement project is to support early identification and intervention at CSMC. Screening data from birthing individuals were included in this study if they were admitted to the postpartum unit or the maternal-fetal care unit after delivery. Our dataset does not include screening data from the prenatal or predelivery time point or from patients who experienced stillbirth. The hospital’s institutional review board reviewed this project and approved the use of deidentified patient data and thus waived the need to obtain patient informed consent.^8,9^ In February 2022, screening for PMAD transitioned from the PHQ-9 to the EPDS, which was originally developed to screen for perinatal depression, but further assesses anxiety symptoms.^66^

### Race and Ethnicity

Race and ethnicity were provided by the patient during intake or later through the patient portal. Patients selected from applicable categories, including African American or Black, American Indian or Alaskan Native, Asian or Native Hawaiian or Other Pacific Islander, White, and other (not further specified). None of the patients in our sample identified as American Indian or Alaskan Native. If a patient selected multiple categories, we categorized them as multiple races. If a patient declined to answer, we categorized them as unknown race but did not exclude their data from analysis due to potential systematic reasons for withholding this information. Patients selected from ethnicity categories of Hispanic or not Hispanic.

### Depression Screening

#### PHQ-9

The PHQ-9 was developed for the dual purpose of establishing tentative diagnoses of depressive episodes and depressive symptom severity.^67^ Each question in the scale had 4 response choices: “not at all,” “several days,” “more than half the days,” and “nearly every day.” Total scores ranged from 0 to 27, which assessed the presence and severity of a depressive episode,^68^ with higher scores indicating greater symptoms of depression.

#### EPDS

The EPDS was developed to screen for PMADs.^69,70^ Patients indicated on a 4-point Likert scale the frequency with which they experienced symptoms (eg, “I have blamed myself unnecessarily when things go wrong”) during the past 7 days. Total scores ranged from 0 to 30, with higher scores indicating greater symptoms of PPD.

### Data Preparation

The target variable was patients’ screening results from either the PHQ-9 or the EPDS, which were dichotomized into low risk (negative) or moderate to high risk (positive) based on the clinical cutoffs used at CSMC, which are more sensitive than research-based cutoffs, to ensure that patients with PMAD are identified and referred for treatment.^9^ Both measures screen for suicidal ideation. Screening positive was determined by endorsement of suicidal ideation or a PHQ-9 score of 5 or higher or a EPDS score of 8 of higher.

### Dataset

The full dataset consisted of 19 790 EHRs. Patients were screened for depression no more than 9 days following delivery. Only the first complete record from each patient was included in the analysis. Data were analyzed separately for patients who received the PHQ-9 and the EPDS. The final dataset consisted of 19 430 unique patients.

### Machine Learning

Deidentified data and codes for model fitting and visualization are available on GitHub.^71^ Model fitting and statistical analyses were conducted with the Python packages, sklearn and scipy, respectively. Statistical tests assume a significance level of α < .05 and 2-tailed tests of hypotheses.

#### Models

Three supervised classification algorithms were fitted to the data: logistic regression, random forest, and extreme gradient boosting. The Box outlines the predictors used to train each model. To determine 1 set of hyperparameters for each model, the dataset was first split into approximately 75% for training, 15% for validation, and 10% for testing using stratified random sampling (stratified by race and outcome). Random undersampling of the training set was used to address issues related to class imbalance. Hyperparameters were fine-tuned using the tree-structured Parzen estimator sampling algorithm from the optuna library, with an objective to maximize the AUROC in the validation set. The test performance (AUROC) was calculated for each model. To provide a more robust evaluation of each set of fine-tuned hyperparameters, each of the 3 models was subsequently fitted assuming the set of fine-tuned hyperparameters and were evaluated using repeated 10-fold cross validation (repeated 10 times). In every fold, random undersampling was implemented on the training split to address issues related to class imbalance. In each of the 100 total iterations, fairness and performance (AUROC) metrics were computed for each race and ethnicity in the testing split.

Box. **Predictor Variables for Machine Learning Models**

**Predictors**

Age
  Birthing patient age at time of delivery
Race
  African American or Black, Asian American and Pacific Islander, Hispanic White, non-Hispanic White (reference group in logistic regression), multiple race, other, and unknown
Ethnicity
  Hispanic, non-Hispanic (reference group in logistic regression), unknown
Marital status
  Married (reference group in logistic regression), single, significant other, domestic partner, married, legally separated, divorced, widowed, unknown
Financial class
  Government (0) or private (1) health insurance
Low birth weight
  Infant birth weight <2500 g
Preterm birth
  Gestational age <36 wk
Delivery method
  Vaginal (0) or cesarean (1)
Neonatal intensive care unit admission
Maternal fetal care unit admission
Gestational preeclampsia
Gestational diabetes
Gestational hypertension
History of anxiety
  Previous diagnosis of anxiety, postpartum anxiety, panic, or posttraumatic stress disorder (at least 1 y prior to delivery)
History of depression
  Previous diagnosis of depression, postpartum depression (at least 1 y prior to delivery)
History of bipolar disorder
  Previous diagnosis of bipolar disorder (at least 1 y prior to delivery)
History of postpartum mental health disturbances
  Previous diagnosis of postpartum mental health disturbance related to another pregnancy (at least 1 y prior to delivery)
Mental health diagnoses
  Previous diagnosis of any psychiatric disorder (at least 1 y prior to delivery),
History of psychotherapeutic medication
  Psychotherapeutic prescription prior to delivery
History of cardiovascular medication

##### Metrics of Algorithmic Fairness

Multiple metrics to measure algorithmic fairness exist in the literature (eg, demographic parity [DP]^72^ and equalized odds^73^). Although Huang et al^65^ operationalized bias as the differences in AUROCs—a metric that jointly evaluates true- and false-positive rates—health disparities in PMAD diagnoses are specifically in underdiagnosis and higher false-negative rates in certain populations. Therefore, the present study evaluated models on DP and false-negative rates between patients in racial and ethnic minority groups and non-Hispanic White patients. Demographic parity is a condition that posits that the predictions of a model ought to be independent of group membership.^74^ However, it may not be an appropriate measure of fairness when there are known disparities in disease prevalence among groups,^74^ as in PMADs.^29,30,31^ Therefore, DP was used for evaluating whether models correctly recovered known variations of PMADs (eg, higher for Black patients than for non-Hispanic White patients). In a classification case, DP was calculated as *DP = P*(ŷ = 1|*g* = *a*) − *P*(ŷ = 1|*g* = *b*), where ŷ indicates the model predictions, and *g* indicates group membership.

#### Additional Bias Mitigation Techniques

Multiple methods have been proposed over the years to mitigate bias in various stages of the machine learning pipeline. These methods may modify properties of the dataset (preprocessing^75,76,77,78^), model (in processing^79^), or predictions (postprocessing^73^) to optimize an objective function (eg, accuracy).

In addition to training models to predict a target variable that is less likely to reflect existing dipartites, we evaluated a preprocessing technique called reweighing.^75^ This method assigns weights to data points in the training dataset to change their contribution to the objective function of the model. The current study calculated sample weights in accordance with the formulation in the study by Kamiran and Calders.^75^

### Statistical Analysis

#### One-Sample *t* Tests

To test the overall direction of potential bias across all baseline models (ie, models without reweighing), 1-sample *t* tests were performed for the DP and differences in false-negative rates from the test sets. The null hypotheses assert that the true differences are equal to zero.

#### Independent Samples *t* Tests

To test the effect of reweighing on the aforementioned metrics, metrics obtained from models without reweighing were compared with those with reweighing using independent samples *t* tests. The null hypotheses assert that the differences between models with or without reweighing are zero.

All analyses were conducted with Python, version 3.11.9, software (Python Software Foundation). A 2-sided value of *P* < .05 was considered statistically significant.

## Results

The final dataset used for model training and evaluation consisted of 11 377 PHQ-9 records and 8658 EPDS records, with a total of 19 430 unique patients between 14 and 59 years of age (mean [SD] age, 34.1 [4.9] years) at the time of their first delivery at CSMC. Among them, 1402 (7%) identified as African American or Black, 2371 (12%) as Asian American and Pacific Islander, 1842 (10%) as Hispanic White, 10 942 (56%) as non-Hispanic White, 606 (3%), as multiple races, 2146 (11%) and as other (not further specified); 121 (<1%) did not provide this information. We differentiated between only non-Hispanic and Hispanic White patients, rather than other groups, because while the sample included other race categories with Hispanic ethnicity, the number of individuals in each group was too small for meaningful analyses. All patients identified as female. During the transition of CSMC from using the PHQ-9 to using the EPDS, 605 patients with complete data received both measures and were included in each set of analyses.

### Prevalence

In our sample, racial and ethnic minority patients were more likely than non-Hispanic White patients to screen positive on both the PHQ-9 (odds ratio, 1.47 [95% CI, 1.23-1.77]) and the EPDS (odds ratio, 1.38 [95% CI, 1.20-1.57]. eTable 2 in Supplement 1 gives prevalence rates for each racial and ethnic group.

### Machine Learning Performances

**Figure 1.**
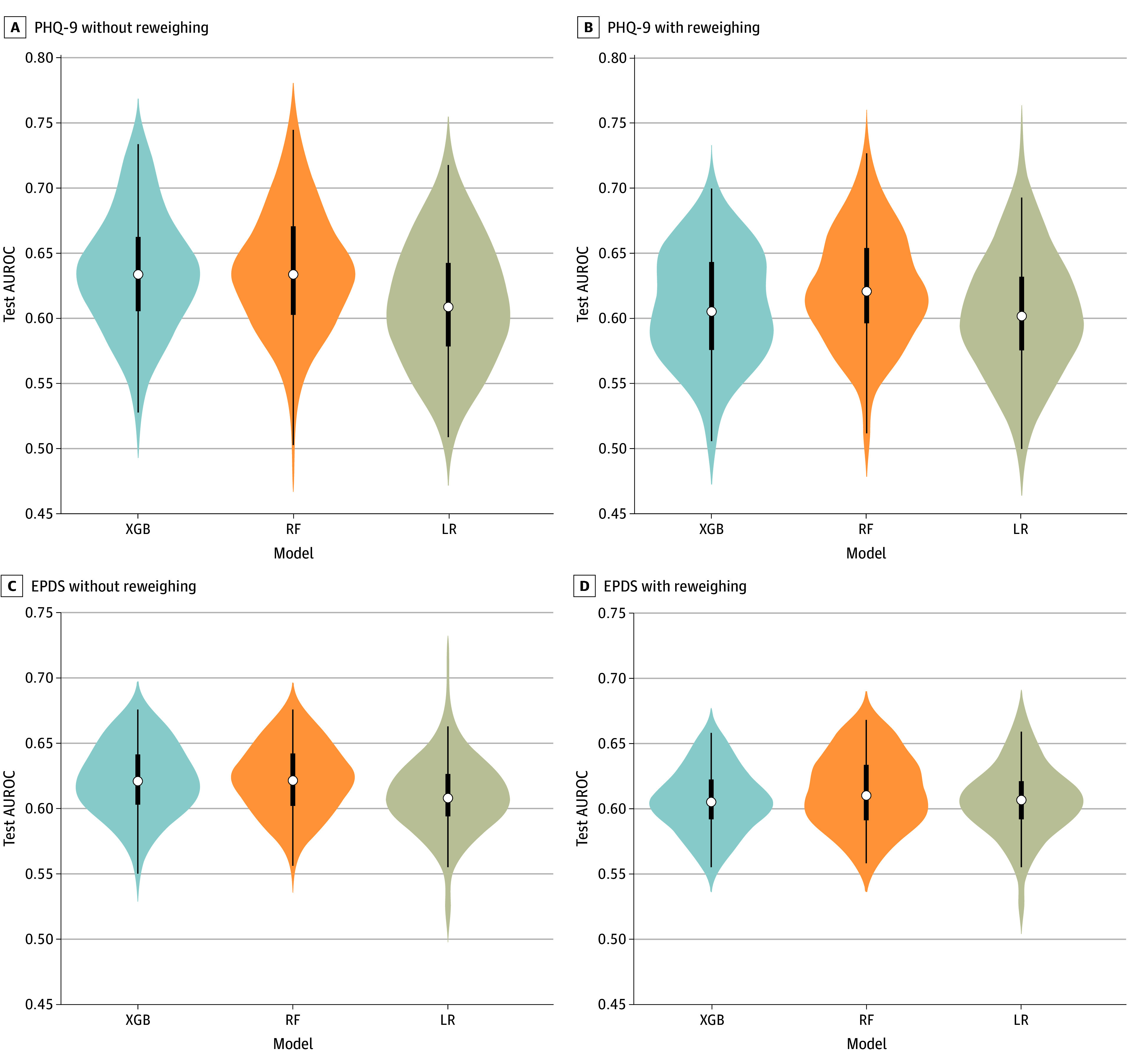
Distribution of the Model Test Areas Under the Receiver Operating Curves (AUROCs) From Repeated 10-Fold Cross Validation The white marker in the middle of each distribution represents the median; thick lines, the 25th and 75th percentiles; and thin lines, minimum and maximum data values. EPDS indicates Edinburgh Postnatal Depression Scale; LR, logistic regression; PHQ-9, Patient Health Questionnaire 9; RF, random forest; and XGB, extreme gradient boosting.

**Figure 2.**
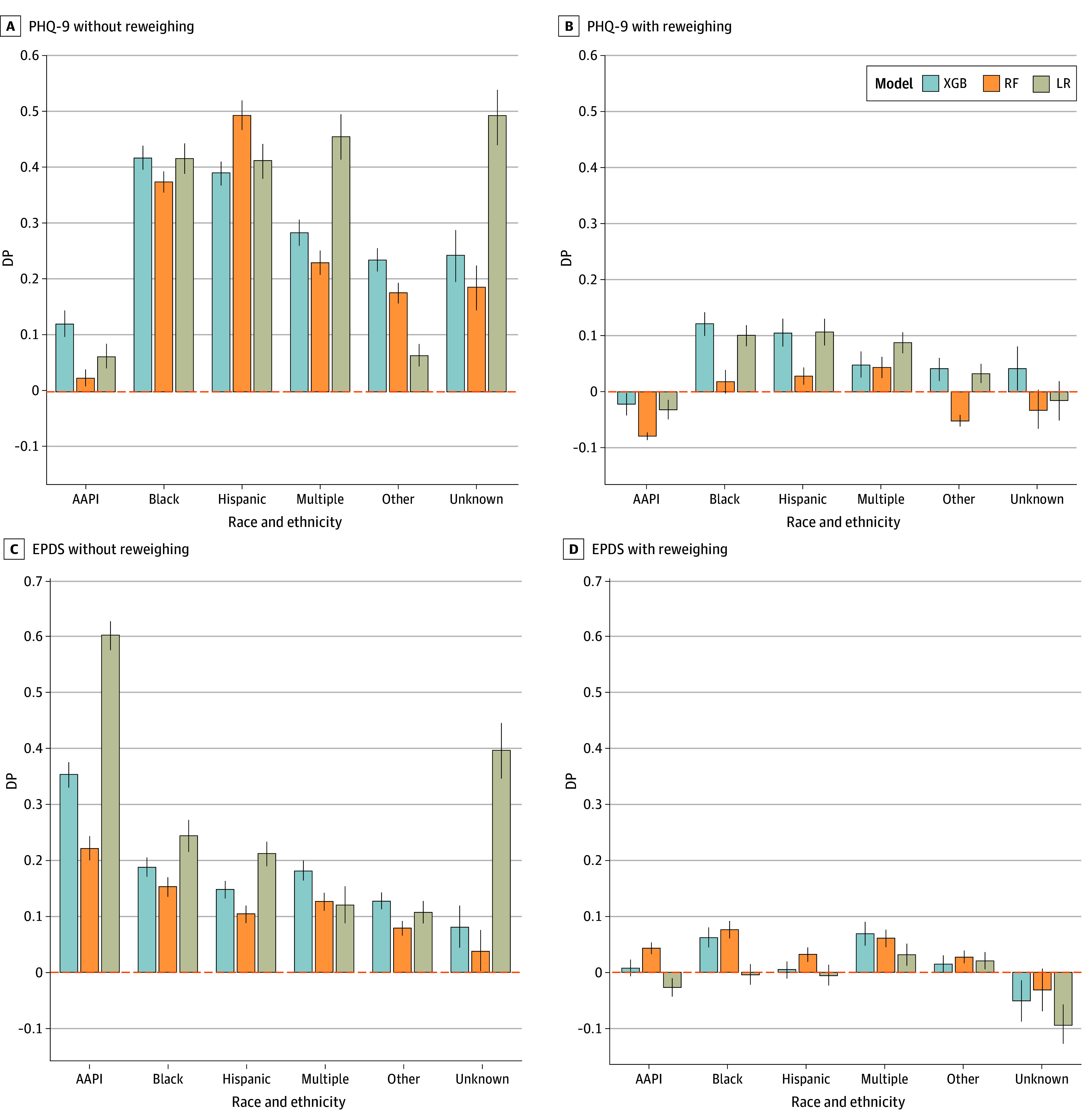
Demographic Parity (DP) for Each Race and Ethnicity Group Relative to Non-Hispanic White Patients The figure collapses across ethnic groups but distinguishes between Hispanic and Non-Hispanic White patients. Error bars represent 95% CIs. AAPI indicates Asian American and Pacific Islander; EPDS, Edinburgh Postnatal Depression Scale; LR, logistic regression; PHQ-9, Patient Health Questionnaire 9; RF, random forest; and XGB, extreme gradient boosting.

**Figure 3.**
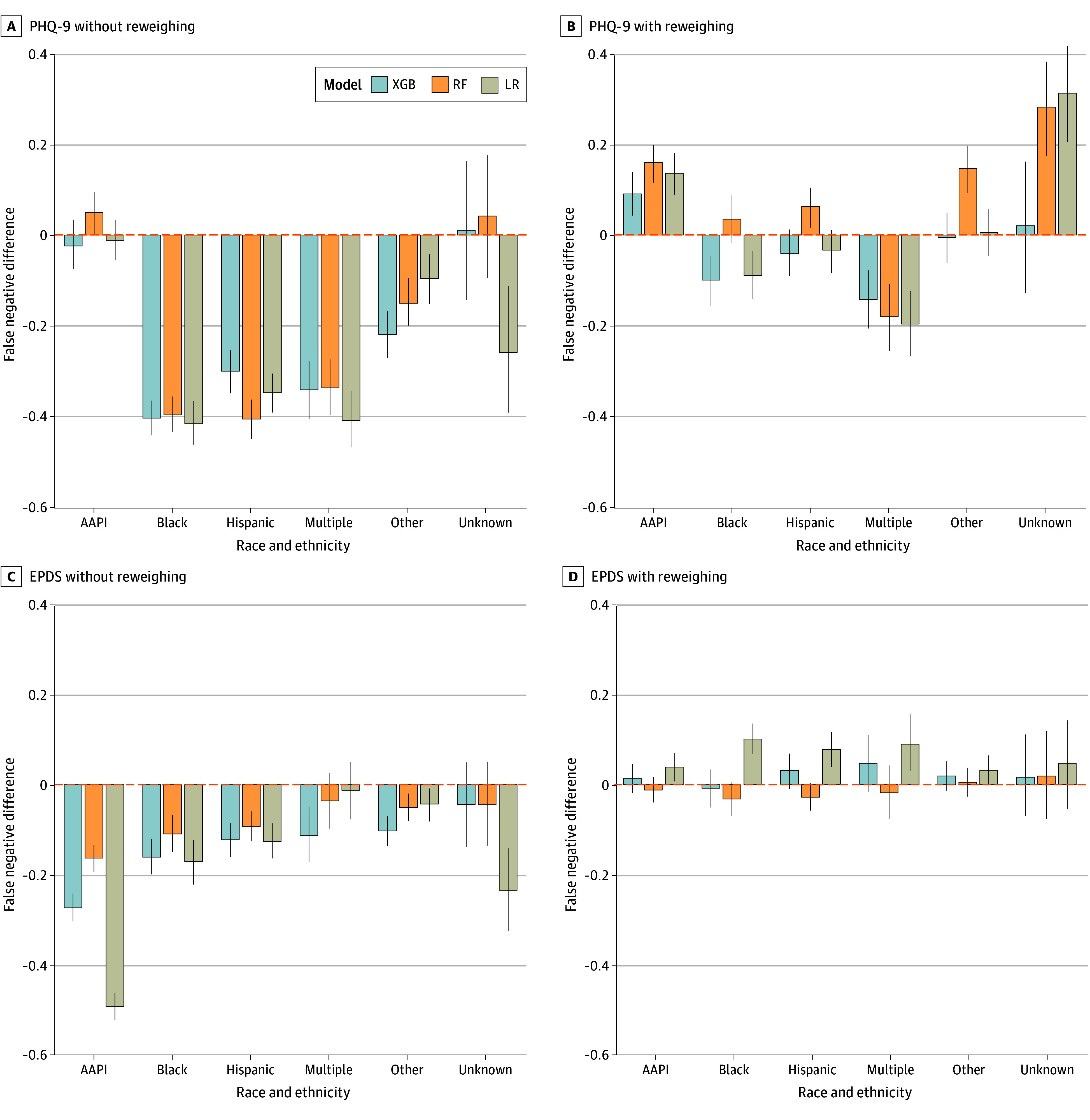
False Negatives for Each Race and Ethnicity Group Relative to Non-Hispanic White Patients The figure collapses across ethnic groups but distinguishes between Hispanic and Non-Hispanic White patients. Error bars represent 95% CIs. AAPI indicates Asian American and Pacific Islander; EPDS, Edinburgh Postnatal Depression Scale; LR, logistic regression; PHQ-9, Patient Health Questionnaire 9; RF, random forest; and XGB, extreme gradient boosting.

The present models were comparable with or outperformed (eTable 3 and eFigures 1-5 in Supplement 1) other studies using similar target and predictor features^64,65^ (mean AUROCs between 0.602 and 0.635). The Table displays the mean test AUROC of each model with (0.610 to 0.635) or without (0.602 to 0.622) reweighing the samples in the training set. Figure 1 displays the AUROCs of the 3 models obtained from 100 unique training-testing splits. Figure 2 and Figure 3 display the DP and differences in false negatives between each racial and ethnic group and non-Hispanic White patients.

**Table.** Mean Test Area Under the Receiver Operating Curve (AUROC)

Scale/reweigh	Mean AUROC (95% CI)		
	Logistic regression	Random forest	Extreme gradient boosting
**PHQ-9**			
No	0.610 (0.601-0.620)	0.635 (0.626-0.644)	0.635 (0.626-0.643)
Yes	0.602 (0.594-0.611)	0.622 (0.613-0.630)	0.609 (0.600-0.617)
**EPDS**			
No	0.611 (0.605-0.616)	0.624 (0.619-0.629)	0.623 (0.618-0.628)
Yes	0.607 (0.602-0.613)	0.614 (0.609-0.620)	0.607 (0.602-0.612)

Abbreviations: EPDS, Edinburgh Postnatal Depression Scale; PHQ-9, Patient Health Questionnaire 9.

### Statistical Analyses for Bias Metrics

#### One-Sample *t* Tests

Without reweighing, the models predicted greater positive rates for racial and ethnic minority patients than for non-Hispanic White patients (DP), (mean DP, 0.238 [95% CI, 0.231-0.244]; *P* < .001). They also displayed lower false negatives for racial and ethnic minorities relative to non-Hispanic White patients (mean difference, −0.184; [95% CI, −0.195 to −0.174]; *P* < .001).

#### Independent Samples *t* Tests

Reweighing seemingly minimized differences in DP. Overall, DP was lower in models with reweighing (mean DP, 0.022 [95% CI, 0.017-0.026]), than models without (see aforementioned values) (*P* < .001). Overall differences in false-negative rates were also lower with reweighing (mean difference, 0.018 [95% CI, 0.008-0.028]) than without (see aforementioned values) (*P* < .001).

Given that non-Hispanic White patients were among the least likely to screen positive in our dataset, their data points were up weighted in the reweighing process. While reweighing data based on observed frequencies appears to minimize differences in false-negative rates between patients from various racial and ethnic groups and non-Hispanic White patients, doing so may perpetuate biases against patients who are not non-Hispanic White (Figure 3).

## Discussion

The present diagnostic study evaluated the ability of machine learning to predict PPD screening results, given commonly available EHR data elements, and emphasized the need to use target variables that are less likely to reflect existing disparities, an issue first raised by Obermeyer et al.^52^ Additionally, given that health disparities in PMADs specifically lie in underdiagnosis and missed diagnoses for racial and ethnic minorities relative to non-Hispanic White patients, the present study operationalized bias as differences in false-negative rates. Together with routine screening, the current results demonstrated how model designs that integrate knowledge of health disparities may limit the potential of an algorithm to exaggerate those disparities. This approach also operationalized bias to be more context-specific, thereby providing a more nuanced understanding of potential algorithmic bias in predictive models of PPD. Unlike prior research, the present models do not perpetuate the same biases against Black and Hispanic patients relative to non-Hispanic White patients. False-negative rates were lower for racial and ethnic minorities, and models were more likely to predict a positive PPD screen for them relative to non-Hispanic White patients (DP). The difference in conclusions between the present study and those from Huang et al^65^ is likely due to different definitions of bias (ie, AUROC in Huang et al^65^ vs false negatives in the present study). Therefore, it may be possible that the best performing model in the study by Huang et al^65^ also had lower false-negative rates for Black and Hispanic patients relative to non-Hispanic White patients, but had higher false-positive rates, yielding lower AUROCs (eFigures 6-8 in Supplement 1).

Compared with studies that predict *ICD* codes and pharmacy records using EHRs and prior screening, the current results and those from similar studies^64,65^ displayed lower performance. The model predictions in the present study did not reproduce historic biases in which racial and ethnic minorities were less likely to receive a positive screen.^55^ Additionally, our results likely corroborate prior findings that demonstrate the predictive power of prior psychometric screening results.^43,44,58,59,60,61,62,63^

We acknowledge that machine learning alone will not resolve issues related to physical and social barriers.^80,81,82,83^ Instead, we propose machine learning as a component of achieving more equitable health care. Achieving completely equitable mental health care will require fundamental restructuring of clinical workflows, establishing more comprehensive mental health services, and improving mental health and implicit bias training for clinical workers. Furthermore, while machine learning tools may support early detection efforts, there may be limited resources after a positive screen, a critically important problem outside the scope of this study.

### Limitations

Ideally, the accuracy of the models would be equal across all patient demographics. However, deterministically reweighing based on expected and observed frequencies in the data can potentially rebias against certain racial and ethnic groups, as observed in our results.

## Conclusions

In this diagnostic study of predictive models of postpartum depression, clinical prediction models trained to predict psychometric screening results from commonly available EHR data elements achieved modest performance and were less likely to widen existing health disparities in PMAD diagnosis and potentially treatment. Future work should explore methods to optimize the weights during training that achieve specific performance and fairness goals.

## Notes

Supplement 1. **eTable 1.** Percentage of patients with records of psychotherapeutic drugs, prescribed prior to delivery, across racial groups **eTable 2.** Counts of patients who received the PHQ-9 and EPDS measures **eTable 3.** Mean test AUROC without reweighing during preprocessing with associated 95% confidence intervals (bootstrapped) **eFigure 1.** Model test AUROC’s across 100 bootstrapped test sets and collapsed across sensitive attributes **eFigure 2.** Feature importance based on mean accuracy decrease for predicting PHQ-9 outcome **eFigure 3.** Feature importance based on mean accuracy for predicting EPDS outcome **eFigure 4.** Demographic parity for each race relative to Non-Hispanic White patients **eFigure 5.** False negative for each race relative to Non-Hispanic White patients **eAppendix 1.** Comparing with Huang et al **eFigure 6.** Test AUROCs for each race relative to Non-Hispanic White patients **eFigure 7.** False positive rates for each race relative to Non-Hispanic White patients **eFigure 8.** True positive (TP) rates for each race relative to Non-Hispanic White patients

Supplement 2.
Data Sharing Statement
