# Supplementary material for: Evaluating Bias-Mitigated Predictive Models of Perinatal Mood and Anxiety Disorders
Source: JAMA Netw Open. 2024 Dec 3;7(12):e2438152. doi: 10.1001/jamanetworkopen.2024.38152 (PMC11615713; doi:10.1001/jamanetworkopen.2024.38152)
Supplement: Supplement 1. — eTable 1. Percentage of patients with records of psychotherapeutic drugs, prescribed prior to delivery, across racial groups eTable 2. Counts of patients who received the PHQ-9 and EPDS measures eTable 3. Mean test AUROC without reweighing during preprocessing with associated 95% confidence intervals (bootstrapped) eFigure 1. Model test AUROC’s across 100 bootstrapped test sets and collapsed across sensitive attributes eFigure 2. Feature importance based on mean accuracy decrease for predicting PHQ-9 outcome eFigure 3. Feature importance based on mean accuracy for predicting EPDS outcome eFigure 4. Demographic parity for each race relative to Non-Hispanic White patients eFigure 5. False negative for each race relative to Non-Hispanic White patients eAppendix 1. Comparing with Huang et al eFigure 6. Test AUROCs for each race relative to Non-Hispanic White patients eFigure 7. False positive rates for each race relative to Non-Hispanic White patients eFigure 8. True positive (TP) rates for each race relative to Non-Hispanic White patients [file jamanetwopen-e2438152-s001.pdf]

Supplementary Online Content

Wong EF, Saini AK, Accortt EE, Wong MS, Moore JH, Bright TJ. Evaluating bias-mitigated predictive models of perinatal mood and anxiety disorders. *JAMA Netw Open*. 2024;7(12):e2438152. doi:10.1001/jamanetworkopen.2024.38152

eTable 1. Percentage of patients with records of psychotherapeutic drugs, prescribed prior to delivery, across racial groups

eTable 2. Counts of patients who received the PHQ-9 and EPDS measures

eTable 3. Mean test AUROC without reweighing during preprocessing with associated 95% confidence intervals (bootstrapped)

eFigure 1. Model test AUROC's across 100 bootstrapped test sets and collapsed across sensitive attributes

eFigure 2. Feature importance based on mean accuracy decrease for predicting PHQ-9 outcome

eFigure 3. Feature importance based on mean accuracy for predicting EPDS outcome

eFigure 4. Demographic parity for each race relative to Non-Hispanic White patients

eFigure 5. False negative for each race relative to Non-Hispanic White patients

eAppendix 1. Comparing with Huang et al

eFigure 6. Test AUROCs for each race relative to Non-Hispanic White patients

eFigure 7. False positive rates for each race relative to Non-Hispanic White patients

eFigure 8. True positive (TP) rates for each race relative to Non-Hispanic White patients

eReference.

This supplementary material has been provided by the authors to give readers additional information about their work.

© 2024 Wong EF et al. *JAMA Network Open*.

eTable 1. Percentage of patients with records of psychotherapeutic drugs, prescribed prior to delivery, across racial groups. Comparison group is non-Hispanic White (19.8%).

Race	Percent (%)	*z*	two-sided *p*
AAPI	9.36	12.01	< .001
Black or African American	10.91	8.02	< .001
Hispanic White	13.08	6.83	< .001
Multiracial	15.68	2.49	.013
Other	11.18	9.43	< .001
Unknown	14.05	1.58	.114

© 2024 Wong EF et al. *JAMA Network Open*.

eTable 2. Counts of patients who received the PHQ-9 and EPDS measures. The proportion of those patients who screened moderate or high on each scale are noted in parentheses.

Race	PHQ-9 (prop. PPD)	EPDS (prop. PPD)
AAPI	1396 (.044)	1071 (.166)
Black or African American	830 (.064)	641 (.131)
Hispanic White	1072 (.064)	832 (.124)
Multiracial	300 (.073)	341 (.109)
Other	1212 (.040)	1003 (.110)
Unknown	59 (.102)	67 (.194)
Non-Hispanic White	6508 (.037)	4703 (.100)
	11 377 (.044)	8658 (.115)

Model Performance
eTable 3 is analogous to **Table 2** in the main paper, and **eFigure 1** is analogous to **Figure 1**. The results presented in **eTable 3** and **eFigure 1** were obtained by evaluating each model across 100 *bootstrapped* test sets.

eTable 3. Mean test AUROC without reweighing during preprocessing with associated 95% confidence intervals (*bootstrapped*); evaluated through 100 *bootstrapped* test sets.

Scale	Reweigh	LR	RF	XGB
PHQ9	No	.668 [.661-.674]	.648 [.640-.656]	.670 [.662-.678]
	Yes	.675 [.668-.681]	.658 [.650-.665]	.654 [.646-.662]
EPDS	No	.635 [.628-.642]	.641 [.634-.647]	.635 [.629-.642]
	Yes	.635 [.629-.642]	.628 [.621-.635]	.615 [.609-.621]

© 2024 Wong EF et al. *JAMA Network Open*.

eFigure 1. Model test AUROC's across 100 *bootstrapped* test sets and collapsed across sensitive attributes.

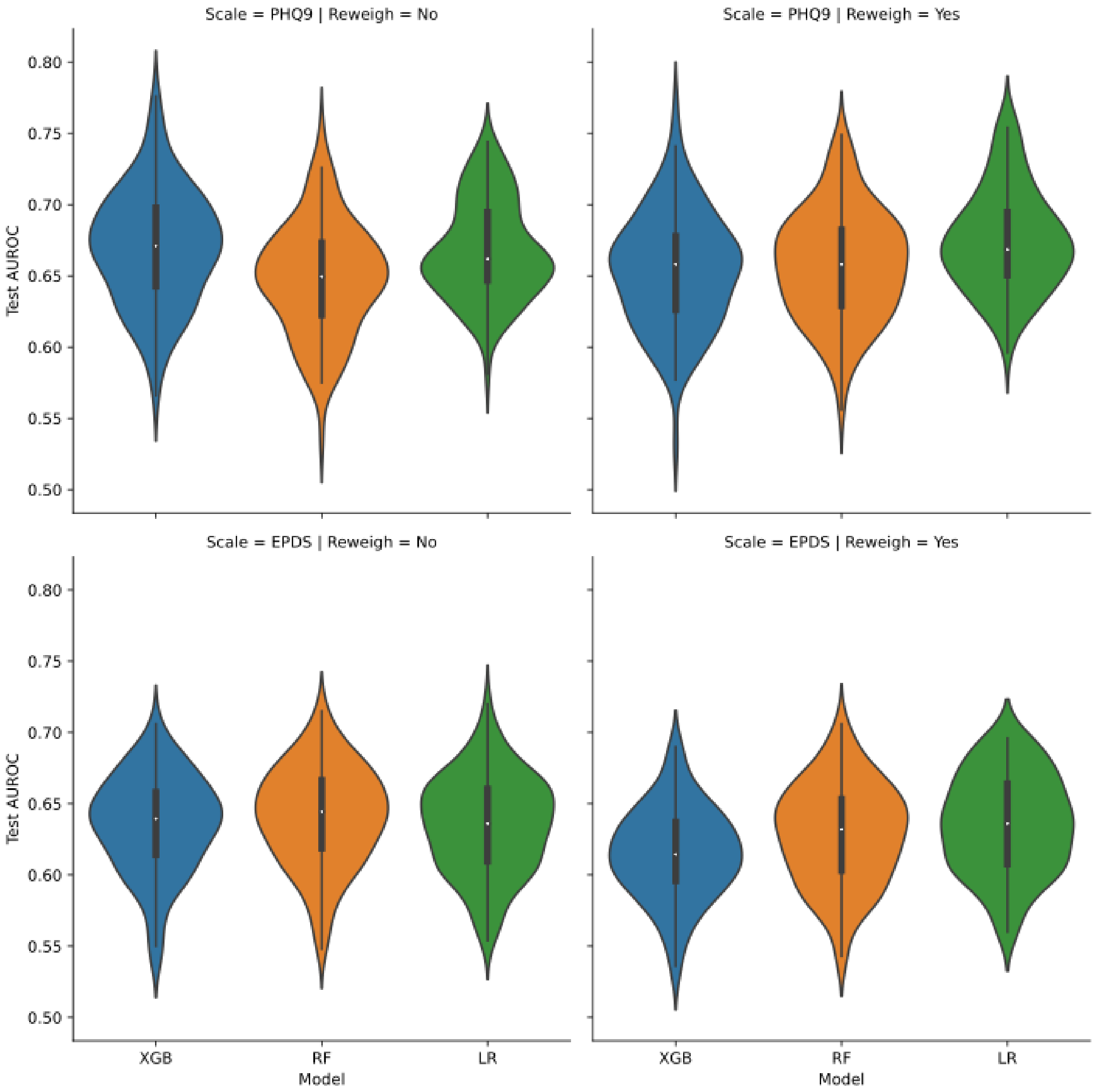

© 2024 Wong EF et al. *JAMA Network Open*.

Variable Importance

Consistent with prior literature, we also found in that a mother's history of mental health diagnoses and treatments were critical. Other social determinants of health were also important features including marital status, race, and ethnicity. **eFigure 2** and **eFigure 3** display variable importance measures obtained from the random forest models without reweighing.

eFigure 2. Feature importance based on mean accuracy decrease for predicting PHQ-9 outcome. Being married (MARITAL_STATUS_Married) was most important.

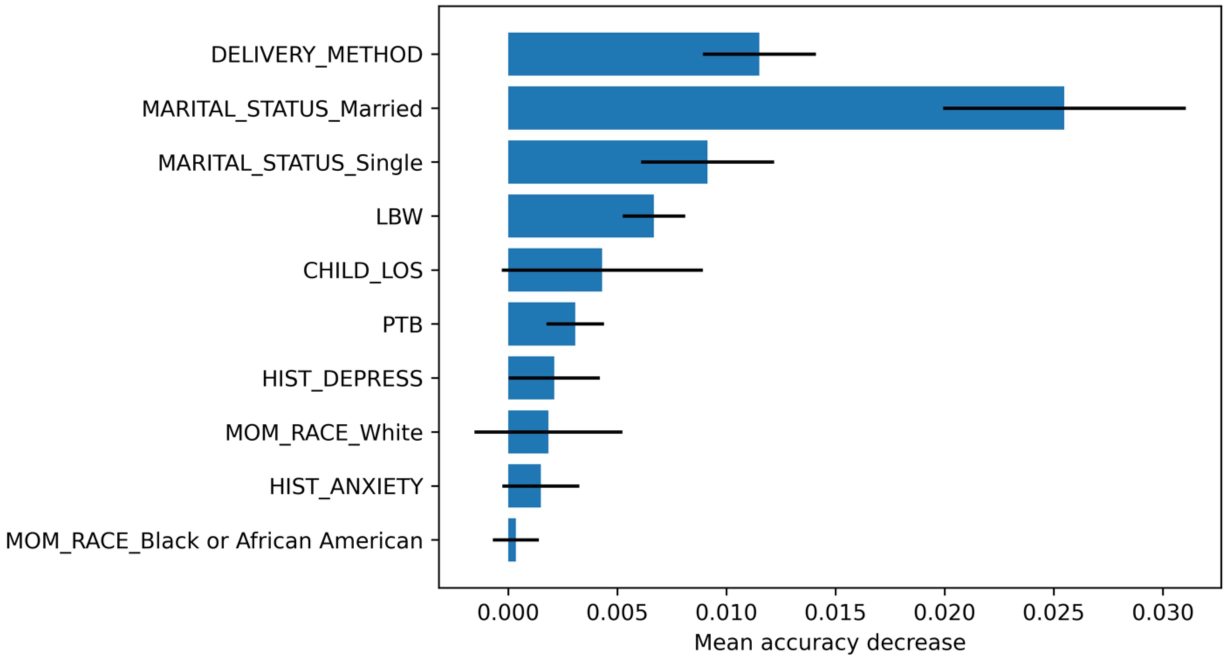

© 2024 Wong EF et al. *JAMA Network Open*.

eFigure 3. Feature importance based on mean accuracy for predicting EPDS outcome. Psychotherapeutic treatment prior to delivery (MED_PSYCH), low infant birth weight (LBW), a history of depression prior to delivery (HIST_DEPRESS), the existence of mental health diagnoses made at least one year prior to delivery (MENTAL_HEALTH_DX_CUTOFF) and being Hispanic were important features.

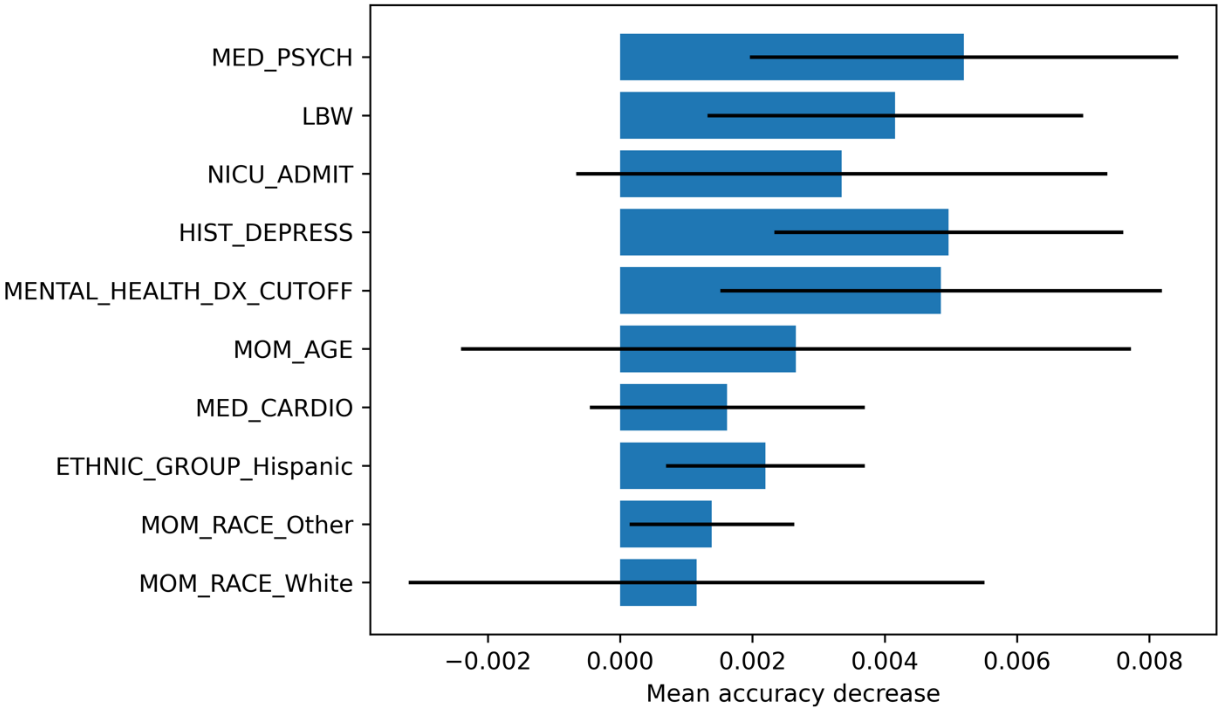

© 2024 Wong EF et al. *JAMA Network Open*.

Evaluations of Algorithmic Bias: Bootstrap
eFigure 4 and **eFigure 5** are analogous to **Figures 2** and **3**, respectively, in the main paper. The models in the two figures below were evaluated across 100 *bootstrapped* test sets.

eFigure 4. Demographic parity for each race relative to Non-Hispanic White patients. The figure collapses across ethnic groups but distinguishes between Hispanic and Non-Hispanic white patients. Error bars represent 95% confidence intervals.

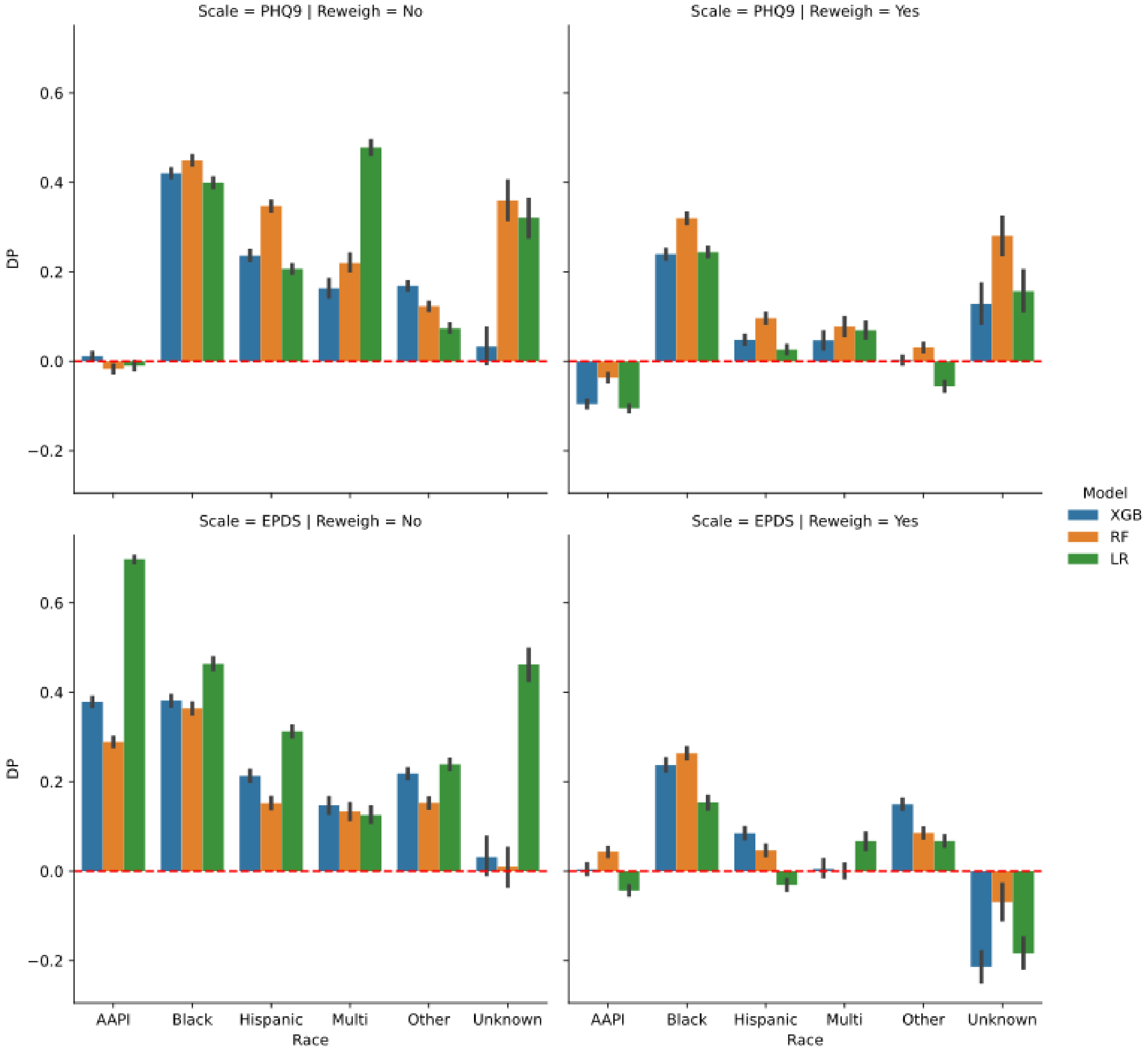

© 2024 Wong EF et al. *JAMA Network Open*.

eFigure 5. False negative for each race relative to Non-Hispanic White patients; bars below the red dotted line indicate lower rates relative to Non-Hispanic White patients. The figure collapses across ethnic groups but distinguishes between Hispanic and Non-Hispanic white patients. Error bars represent 95% confidence intervals.

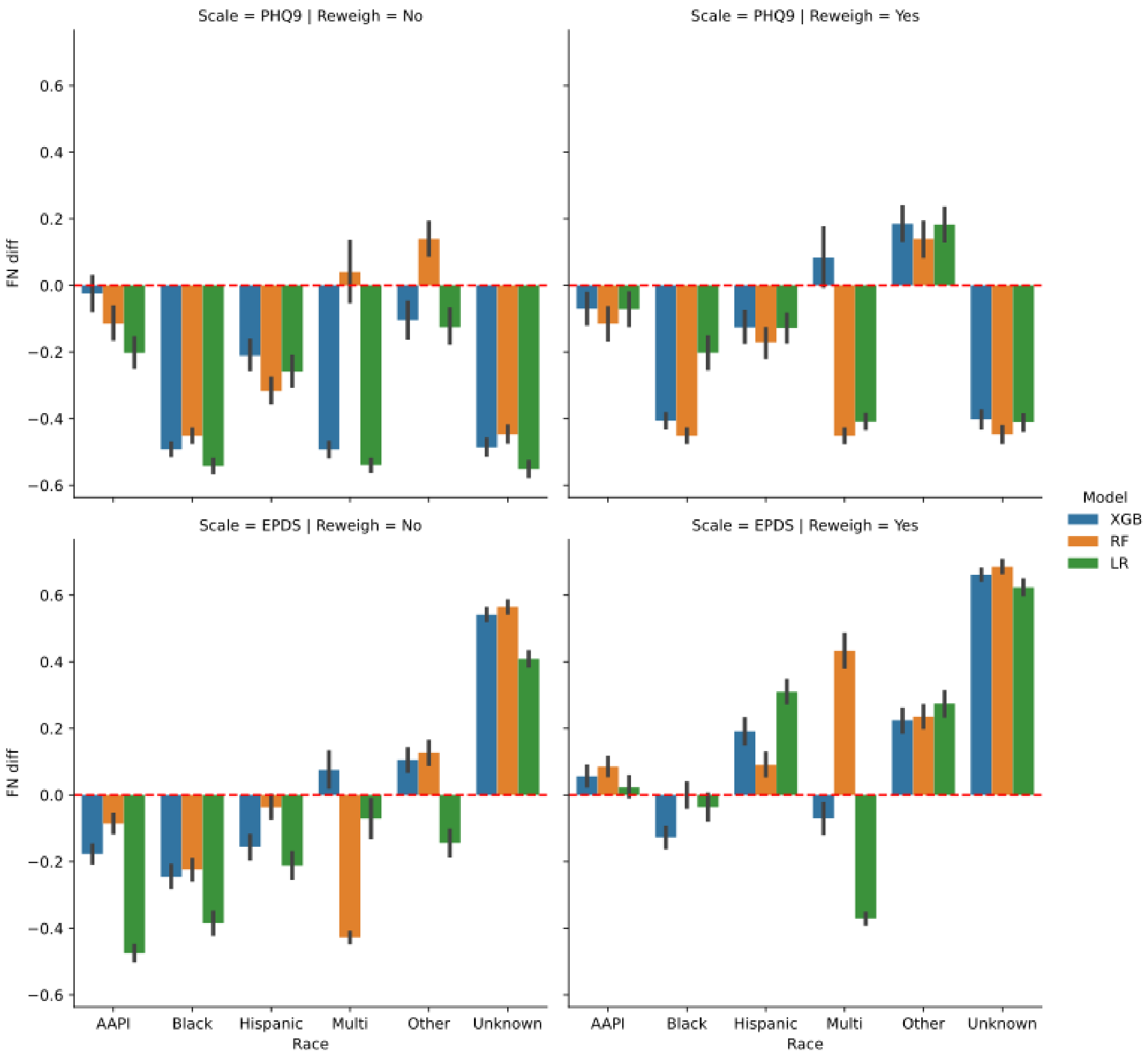

© 2024 Wong EF et al. *JAMA Network Open*.

eAppendix 1. Comparing with Huang et al.[1]

All the results below follow from the *repeated 10-fold cross validation* described in the main paper. The following results address the discrepancy between the conclusions in the main paper and Huang et al[1]. As discussed in the main paper, the discrepancy in conclusions is likely due to differences in how algorithmic bias was defined between the two studies. While our study measured algorithmic bias as differences in false negative rates between racial minorities and non-Hispanic White patients, Huang et al.[1] defined algorithmic bias as lower AUROCs for racial minorities relative to non-Hispanic White patients.

While the stated conclusions differ, the results between the two studies may be similar. AUROC is a function of true and false positives such that higher true positive rates and lower false positive rates yield a higher AUROC value. While the models in our study did display lower AUROC values for ethnic minorities relative to non-Hispanic white patients overall **(**collapsing across all conditions: Model, Reweighing), $M_{diff} = -0.02$, $t(6659) = 9.36$, two-tailed $p < .001$ (see **eFigure 6**), the lower overall AUROC values are likely due to the higher false positive rates for those groups, $M_{diff} = 0.13$, $t(7199) = 53.06$, two-tailed $p < .001$ (see **eFigure 7**). The higher false positive rates for racial minorities indicate that the models were more likely to predict a positive screen for racial minorities than for non-Hispanic White patients. Simultaneously, the true positive rates for racial minorities were higher than for non-Hispanic White patients, $M_{diff} = 0.08$, $t(6659) = 21.70$, two-tailed $p < .001$ (see **eFigure 8**).

© 2024 Wong EF et al. *JAMA Network Open*.

eFigure 6. Test AUROCs for each race relative to Non-Hispanic White patients. Bars below the red dotted line indicate lower values relative to Non-Hispanic White patients. The figure collapses across ethnic groups but distinguishes between Hispanic and Non-Hispanic white patients. Error bars represent 95% confidence intervals.

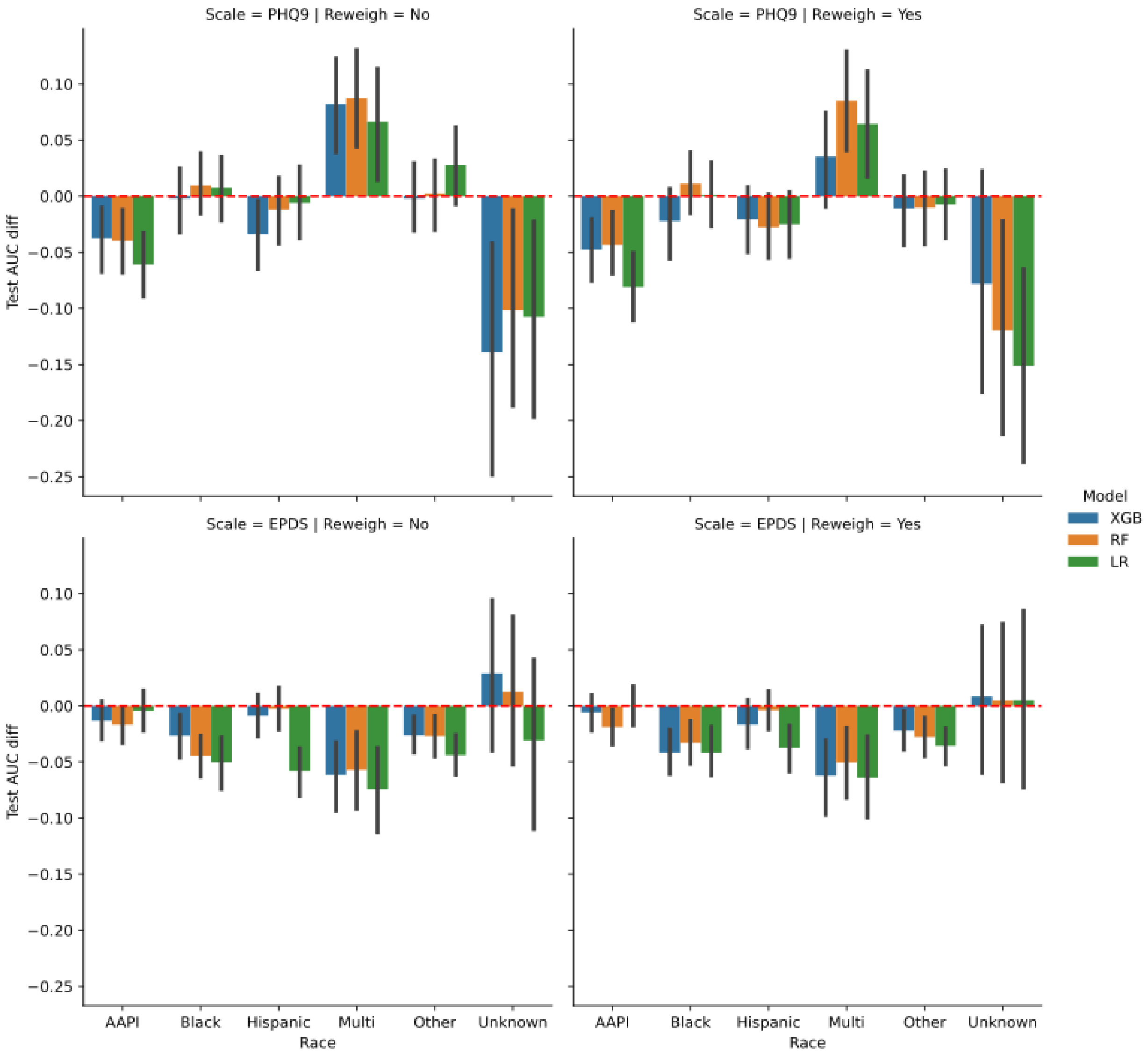

© 2024 Wong EF et al. *JAMA Network Open*.

eFigure 7. False positive rates for each race relative to Non-Hispanic White patients. Bars above the red dotted line indicate higher rates relative to Non-Hispanic White patients. The figure collapses across ethnic groups but distinguishes between Hispanic and Non-Hispanic white patients. Error bars represent 95% confidence intervals.

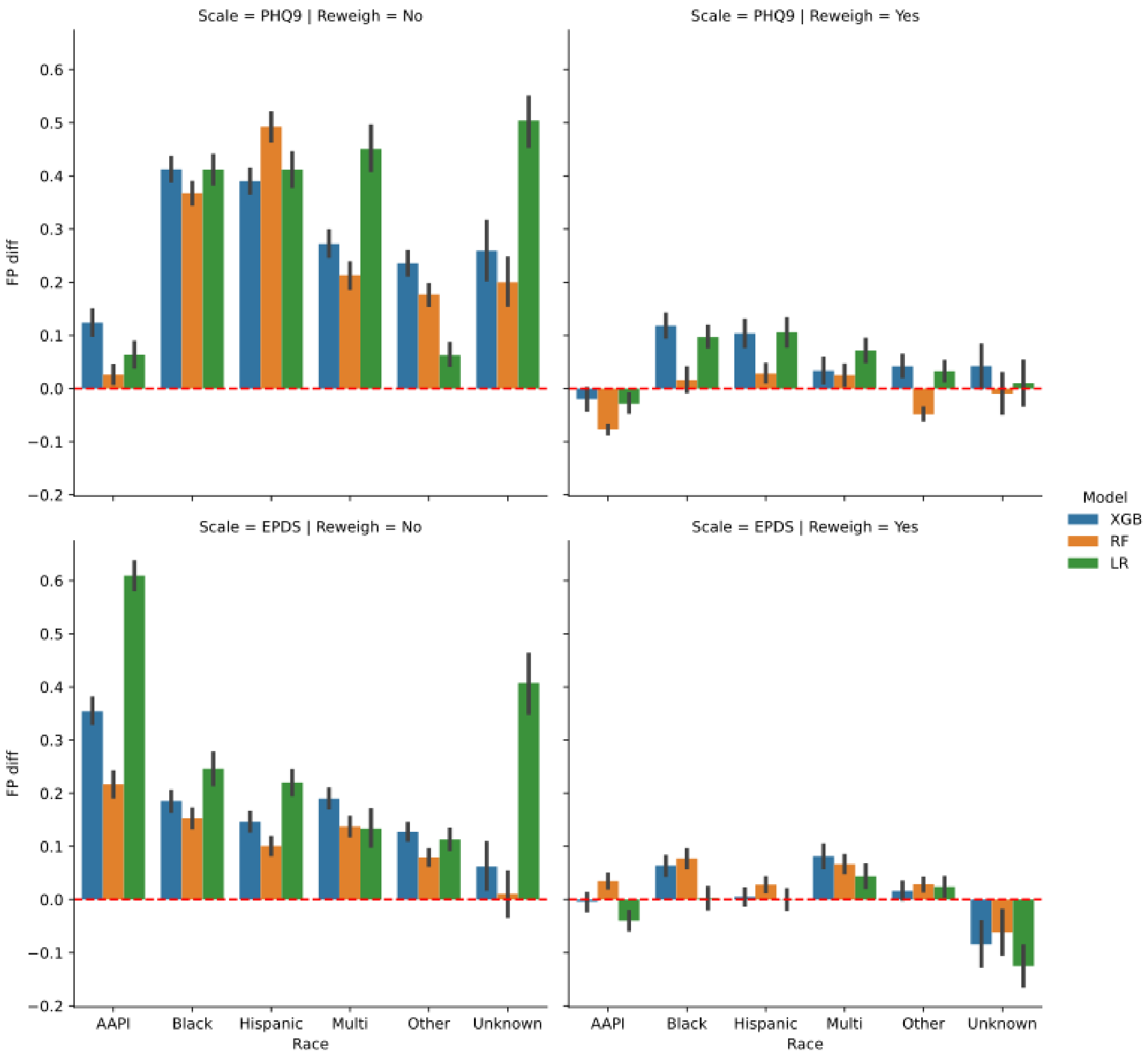

© 2024 Wong EF et al. *JAMA Network Open*.

eFigure 8. True positive (TP) rates for each race relative to Non-Hispanic White patients. Bars above the red dotted line indicate higher rates relative to Non-Hispanic White patients. The figure collapses across ethnic groups but distinguishes between Hispanic and Non-Hispanic white patients. Error bars represent 95% confidence intervals.

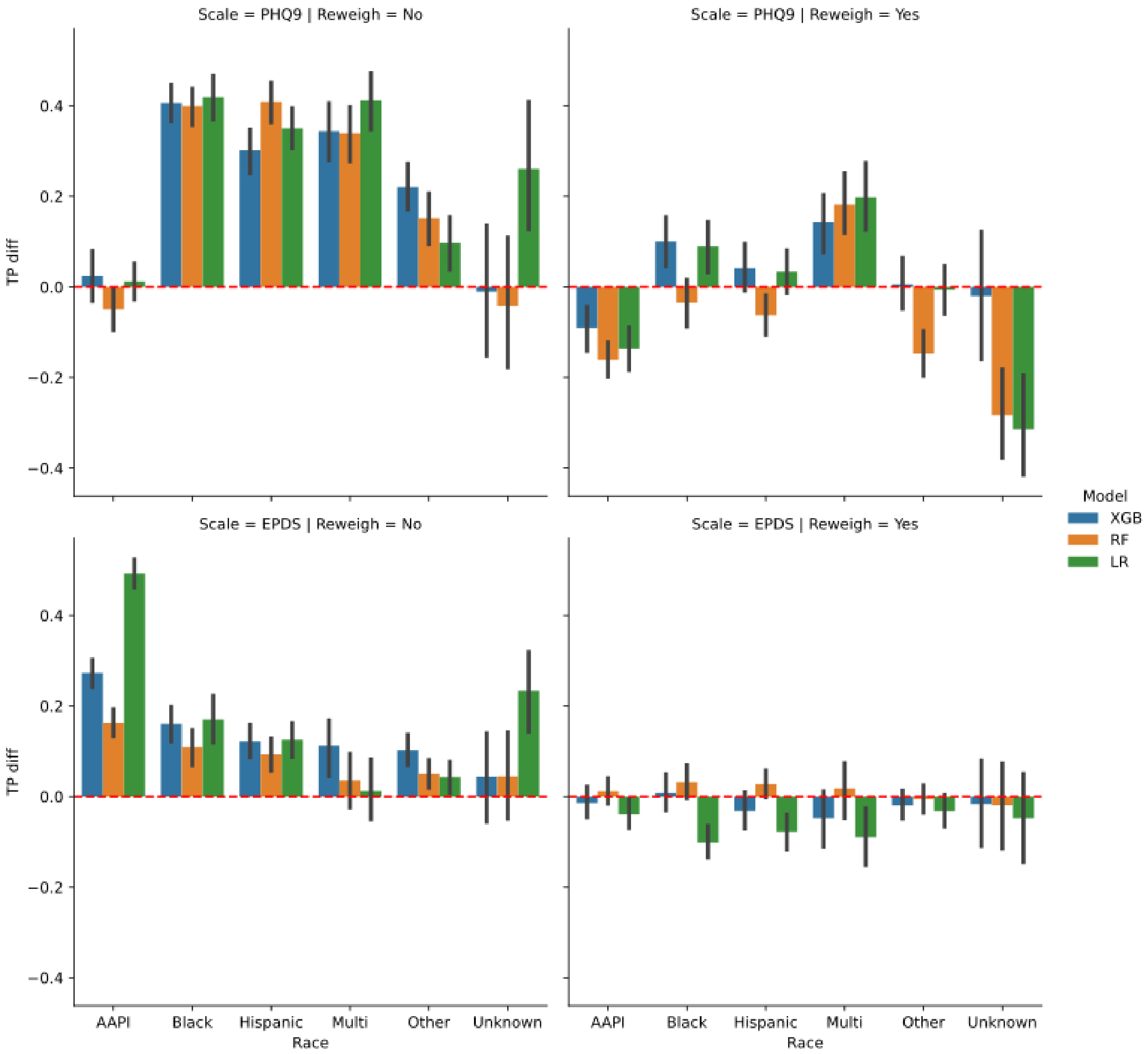

© 2024 Wong EF et al. *JAMA Network Open*.

eReference

1. Huang Y, Alvernaz S, Kim SJ, Maki P, Dai Y, Bernabé BP. Predicting prenatal depression and assessing model bias using machine learning models. *medRxiv*. Published online 2023.

© 2024 Wong EF et al. *JAMA Network Open*.
