# Supplementary material for: Evaluating Bias-Mitigated Predictive Models of Perinatal Mood and Anxiety Disorders
Source: JAMA Netw Open. 2024 Dec 3;7(12):e2438152. doi: 10.1001/jamanetworkopen.2024.38152 (PMC11615713; doi:10.1001/jamanetworkopen.2024.38152)
Supplement: Supplement 2. — Data Sharing Statement [file jamanetwopen-e2438152-s002.pdf]

Data Sharing Statement

Wong. Evaluating Bias-Mitigated Predictive Models of Perinatal Mood and Anxiety Disorders. *JAMA Netw Open*. Published December 03, 2024. doi:10.1001/jamanetworkopen.2024.38152

Data
Data available: Yes
Data types: Deidentified participant data
How to access data: If accepted for publication, the de-identified patient data will be posted to the first author's GitHub that is linked in the paper.
When available: With publication

Supporting Documents
Document types: Statistical/analytic code
How to access documents: All statistical/analytic code has been uploaded to the first author's GitHub that is linked in the paper; available immediately.
When available: beginning date: 04-29-2024

Additional Information
Who can access the data: The code is available to the public. The data will also be made available to the public upon publication.
Types of analyses: All analyses and demographics reported in the main paper and supplemental materials.
Mechanisms of data availability: Without investigator support.
